# Crossmodal Pitch−Luminance Association in Tortoises

**DOI:** 10.1111/nyas.70063

**Published:** 2025-09-16

**Authors:** Maria Loconsole, Beatrice Malaman, Gionata Stancher, Elisabetta Versace

**Affiliations:** ^1^ Department of General Psychology University of Padova Padova Italy; ^2^ Rovereto Civic Museum Foundation Rovereto Italy; ^3^ Department of Biological and Experimental Psychology, School of Biological and Behavioural Sciences Queen Mary University of London London UK

**Keywords:** comparative psychology, crossmodal associations, crossmodal correspondences, pitch−luminance association, reptile cognition, spontaneous choice, *Testudo hermanni*

## Abstract

Crossmodal associations—spontaneous links between sensory modalities—are widely observed in humans. Similar associations have also been found in chimpanzees, monkeys, dogs, chickens, and tortoises, suggesting a shared cognitive strategy based on environmental correlations or a similar nervous system architecture. Alternatively, or complementarily, these associations may arise from learned, species‐specific occurrences. Humans and chimpanzees, for instance, pair higher‐pitched sounds with white shapes and lower‐pitched sounds with black shapes, while baboons and chickens show no preference. Here, we provide novel evidence of pitch−luminance association in a nonvocal reptile, the Hermann's tortoise (*Testudo hermanni*). We studied tortoises in a spontaneous food‐searching task. After hearing a relative higher‐pitched (700 Hz) versus lower‐pitched (450 Hz) sound, animals could choose to search for food behind either a light‐ or dark‐colored wall. Tortoises consistently preferred the white wall with higher‐pitch sounds and the black wall with lower‐pitch sounds, resembling spontaneous associations seen in humans and chimpanzees. Evidence of pitch–luminance association in tortoises suggests that phylogenetically distant species may share similar perceptual organization or internalize similar statistical regularities, shifting the question toward whether such associations reflect homology or convergent evolution.

## Graphical abstract

Hermann's tortoises matched high‐pitched sounds with white and low‐pitched sounds with black in a spontaneous choice task. This mirrors pitch‐luminance associations seen in humans and chimpanzees. The findings suggest that such crossmodal associations may arise from shared perceptual processes across distantly related species, pointing to possible evolutionary or environmental origins of this phenomenon.

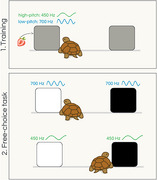

## 1 Introduction

Despite the segregation of sensory receptors, perceptual experiences across modalities are closely interconnected: for example, higher‐pitched sounds tend to bias individuals toward associating them with small, light‐colored objects, whereas lower‐pitched sounds bias responses toward large, dark‐colored ones [1]. Tracing back to von Schiller's pioneering study demonstrating that fish swim toward visual brightness levels associated with specific odors [2]—extending Hornbostel's findings on olfactory‐brightness correspondences in humans [3]—crossmodal correspondences have since been observed in a wide range of species, including chimpanzees [4], rhesus monkeys [5], dogs [6, 7], chickens [8], and tortoises [9], replicating crossmodal effects previously documented in humans. Altogether, these findings suggest that crossmodal associations may represent a widespread cognitive strategy, potentially rooted in the ability to detect and interpret natural correlations across sensory modalities or reflecting similarities in the neural organization/coding [1, 10]. This shared capacity could reflect an evolutionary advantage in processing multisensory information, enhancing perception and decision‐making across diverse ecological contexts. Alternatively, or complementarily, crossmodal associations may stem from learning processes and species‐specific cognitive skills shaped by ecological and social contexts [11].

Different theories have been put forward to explain the presence of crossmodal associations [1, 11, 12]. According to one prominent hypothesis, crossmodal associations arise from the internalization of natural co‐occurrences, enabling animals to predict the most likely outcomes and adjust their responses accordingly. For instance, as body size influences pitch vocalizations, toads use this acoustic information to effectively judge the size of rivals [13], and both dogs and humans can infer the size of another dog from their growls [14, 15]. Supporting this view, evidence from pre verbal infants [16, 17] and precocial species such as the domestic chicken [8, 18] suggests that some crossmodal biases may reflect early emerging perceptual predispositions, possibly shaped by evolutionary pressures to detect meaningful cross‐sensory correlations. Notably, pitch and luminance may not be directly or consistently linked in the natural world—darker objects do not inherently produce lower‐pitched sounds. Some authors argue that such correspondences could instead arise from early developing neural linkages between sensory areas [19]. However, an alternative ecological account posits an indirect correlation, similar to the light‐from‐above prior [11, 20]: in most environments, illumination comes from above, making objects located higher—often smaller and more likely to produce higher‐pitched sounds—appear brighter. This would create a statistical association between brightness, pitch, and size, potentially supporting the tortoises’ observed mappings in this and previous studies.

The ability to match sounds with stimuli from other sensory modalities could have facilitated the evolution of speech [21], as shown, for instance, by the ability of macaques to match facial expressions and sounds [22]. Recent results from primates have also been interpreted as a potential link between human pitch−luminance associations and the evolution of language. Humans [4, 23] and chimpanzees [4] reliably associate higher‐pitched sounds with white shapes and lower‐pitched sounds with black shapes. When tested in a categorization task of white and black shapes, subjects were faster in responding when hearing a background sound congruent to the pitch−luminance association (i.e., higher‐pitched when responding to white, and lower‐pitched when responding to black). In contrast, baboons tested in the same categorization task did not show any consistent preference [23]. Moreover, 3‐day‐old domestic chickens [24] tested in a free‐choice task, where they had to choose between a black or a white panel to locate a food reward, did not show any bias related to higher‐ or lower‐pitched background sounds, indicating that this association may not be universal across all species. Comparative evidence of spontaneous pitch−luminance associations in species that do not depend on vocal communication would confirm a role of crossmodal associations beyond language evolution [23].

Here, we propose the Hermann's tortoise (*Testudo hermanni*) as a suitable model species for the study of spontaneous pitch−luminance association. Hermann's tortoises are solitary and nonvocal, allowing us to test whether pitch–luminance associations are intrinsic and independent of language or social learning. The absence of such associations in chicks and baboons—both vocal and social species—suggests that vocal communication is not a sufficient condition for these biases to emerge, although it remains unclear whether it may still be a necessary one. Additionally, tortoises are highly motivated by food, which enables effective training using food rewards—a method that can be challenging in other reptiles due to their lower metabolic rates and infrequent feeding compared to mammals and birds [25, 26]. Finally, previous research has demonstrated that tortoises are capable of basic cross‐modal processing, as they spontaneously associated auditory pitch with visual size in a choice task [9]. This positions the current study as a natural progression in exploring a different crossmodal correspondence: pitch–luminance. While both studies use pitch as the auditory dimension, pitch–luminance associations have shown inconsistent results across species—failing, for instance, in baboons [23] and poultry chicks [24]. Given that tortoises successfully matched pitch to size, we hypothesized they might also show a pitch–luminance correspondence. If they failed, this would suggest that this particular association may be less general or more cognitively demanding. If they succeeded, as our findings suggest, it may prompt a re‐evaluation of why certain species (e.g., primates, birds) do not show the effect.

**FIGURE 1:**
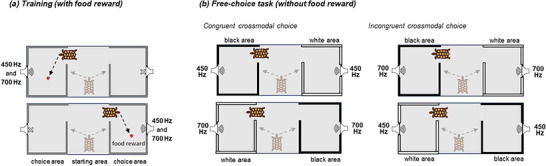
The experimental arena and procedures. The arena is divided into three separate areas: the animal is placed into the central starting area and is free to enter each of the two side areas (choice areas) via a small opening on the side. (a) In each training trial, a piece of strawberry was placed close to the playing loudspeaker. The loudspeaker alternated between higher (700 Hz) and lower (450 Hz) pitch sounds, each lasting 10 s. The arena was dyed all gray. At training, tortoises learned to approach the source of the sound to find the reward. (b) At test, tortoises received no reward. Both speakers played the same pitch sound (either higher or lower). Each choice area can be identified by the color of the walls (either white or black). We hypothesized a preference for the white area in the higher‐pitch sound condition and a preference for the black area in the lower‐pitch sound condition (congruent crossmodal choice) rather than the opposite (incongruent crossmodal choice).

Here, we tested 18 Hermann's tortoises in a food‐searching task comprising 16 consecutive trials where the animals could spontaneously choose to search for food behind either a white (high luminance) or a black (low luminance) wall (Figure 1). During each trial, the same background sound was played simultaneously behind the two doors, either a higher‐pitched (700 Hz) or lower‐pitched (450 Hz) sound. In these unrewarded trials, tortoises showed a significant tendency to choose the white door when hearing the higher‐pitch sound, and the black door when hearing the lower‐pitch sound. Thus, while the procedures differ across species, the observed association is consistent with findings in humans and chimpanzees [4]. Together with mounting evidence of crossmodal associations in different taxa [6, 7, 8], data from a reptile suggest the idea of the widespread presence of spontaneous crossmodal associations as a predisposed and language‐independent mechanism, in line with natural co‐occurrences present in the environment.

## 2 Materials and Methods

### 2.1 Subjects

We tested 18 adult male Hermann's tortoises aged between 15 and 30 years. Tortoises were housed at Sperimentarea (Rovereto Civic Museum Foundation, Italy) in a semi‐free environment, consisting of two open fields of approximately 10×12 meters each, with natural vegetation delimited by metal fences, where the animals lived in same‐sex groups with free access to food and water. These animals are usually brought to Sperimentarea by the local authorities, either after being confiscated or found abandoned. Since tortoises are not endemic in this area, we expect all or most of them to be born in captivity.

The experimental subjects were temporarily housed in a separate area within the main facility, where they had free access to fresh water and were regularly fed lettuce and herbs. To avoid disturbing egg‐laying in females, only male tortoises were included in the study. We used a novel group of subjects housed in the area adjacent to that used in the previous study on pitch‐size correspondences, ensuring similar environmental conditions while involving different individuals. To facilitate individual identification, we documented each tortoise with photographs of both the carapace and plastron. These images reveal distinctive patterns unique to each individual, a method supported by previous research demonstrating the reliability of plastron and carapace patterns for individual recognition in tortoises [27, 28]. This documentation will also aid in deliberately retesting or avoiding the same subjects in future studies.

To avoid environmental confounds, animals were tested individually in a shed separated from their living environment. The experiments were carried out in July−August 2024, during the period of highest activity (tortoises fall into hibernation approximately from November to April).

This study complied with all applicable national and European laws concerning the use of animals in research. All the procedures were examined and approved by the Rovereto Civic Museum Foundation Ethical Review Committee (Prot. n. 0000132 dd. April 12, 2023).

### 2.2 Experimental Arena and Stimuli

The experimental arena consisted of a wooden corridor (50 cm high) divided into three separate areas: a central area (50×50 cm) that served as the animal's starting point, and two choice areas (35×50 cm), each accessible through a small opening in the walls (25×10 cm) on either side of the starting area. On each of the two short sides of the arena, we located a loudspeaker (Extreme Flat Panel Speaker with Amplifier system, model P‐188) to play the auditory stimuli. Mounted above the arena, there was a 400 W halogen lamp and a video camera (Microsoft HD) for recording the tests. Auditory stimuli consisted of a 10‐s higher‐pitch (700 Hz) sound, and a 10‐s lower‐pitch (450 Hz) sound, prepared by using the software Audacity, setting an amplitude of 65 dB. Both frequencies lie within the auditory range of this species, which is known to respond to airborne sounds in the range of 10–940 Hz [29, 30]. Additionally, a previous work on pitch‐size correspondence [9] that employed a similar methodology showed that tortoises are sensitive and responsive to these two frequencies.

### 2.3 Training

The experiment started with a familiarization and training procedure aimed at acquainting the subjects with a neutral gray arena and teaching them to reach the source of the sound to find a palatable reward, a small piece of strawberry. In this stage, the arena's floor and walls were lined with 290 g opaque gray contact paper (opaque polyvinyl chloride). Each tortoise underwent several training sessions, from a minimum of 3 to a maximum of 6. Each session lasted approximately 30 min and consisted of a series of consecutive trials. If the subject reached a maximum of 24 trials, or exceeded 30 min of training, the session ended, and the subject was allowed to rest for a minimum of 2 h. In each trial, only one speaker was active, playing an alternation of the higher‐ and lower‐pitch sounds spaced out by 1 s of silence. The position of the playing speaker was counterbalanced between trials.

Training animals to the presence of food hidden behind a door, close to the sound source, was achieved through a shaping procedure. The animal was placed into a starting box in the center of the empty arena with the strawberry close to the playing loudspeaker. Next, the two central walls (each with a small lateral opening that allowed access behind the wall) were added, creating three distinct areas. At this stage, the strawberry was still visible at the entrance of the correct opening. Finally, the strawberry was hidden behind the wall, so the tortoises could rely only on the auditory cue to locate the correct area to enter. The position of the reward (left or right opening with respect to the tortoise's position) was counterbalanced between trials. Specifically, to minimize the risk of tortoises adopting a lose‐shift or alternate‐direction strategy, we ensured that the rewarded side was never the same for more than two consecutive trials, preventing the establishment of a predictable pattern. The first/second sound heard (alternation of lower–higher or higher–lower pitch sounds) was counterbalanced between trials.

When the subjects entered the correct area (area with the sound and strawberry), they were allowed to eat the food reward. Then, they were removed from the arena, and a new trial was started. When the subjects entered the incorrect area, they were immediately removed from the arena without the possibility to enter the other area, and a new trial was started.

Each subject moved to the next step of the shaping procedure when they responded correctly to six consecutive trials out of eight, within 3 min per trial. If this criterion was not met, the last step of the shaping procedure was repeated.

### 2.4 Test

The test was always conducted in a single, separate session from the training. Before undergoing the test, each subject went through a refresh phase consisting of four additional training trials. Then, each animal underwent 16 consecutive test trials. We selected this number of test trials based on evidence from our previous study [9] using the same methodology, in which animals began to show signs of extinction—that is, a decline in the learned behavior [31]—after approximately 16 unrewarded trials. This allowed us to collect reliable data while minimizing the risk of behavioral extinction and potential stress.

During the test, the starting area remained the same as in training, but the adjacent walls were replaced with colored panels. Each wall consisted of a large cardboard partition that could be slid into a side‐mounted track, allowing us to easily swap the gray training walls with a white (170 hue, 0 saturation, 255 lightness) and a black (170 hue, 0 saturation, 0 lightness) one. Each wall was either black or white and included an opening leading to an identically colored (achromatic, black or white) compartment behind it. Notably, this test session marked the first time the tortoises were exposed to the black and white walls, ensuring that their responses were not influenced by prior experience with these specific stimuli. This approach minimizes potential confounding factors associated with stimulus familiarity and extensive training, allowing us to assess spontaneous crossmodal associations more effectively [11].

During testing, both speakers were active, meaning that the source of the sound was no longer a reliable cue for locating the food reward (Figure 1). For each trial, the speakers played a repetition of the same sound, either the higher‐pitch or the lower‐pitch, with 1 s of silence between each repetition. The position of the white and black walls (i.e., left or right), as well as the pitch of the sound, were pseudo‐randomly alternated between trials. No food reward was present during the test to rule out possible olfactory confounding. After each trial, once the tortoise made a choice, it was gently removed from the arena without being allowed to inspect the unchosen area and placed in a transport box adjacent to the arena while we prepared the setup for the subsequent trial. The preparation time between trials was approximately 30 s, after which the tortoise was returned to the arena to begin the next trial.

We hypothesized that if tortoises rely on predisposed crossmodal associations to solve the task, they should choose the white area more often when hearing the higher‐pitch sound, and the black one when hearing the lower‐pitch sound [4].

### 2.5 Quantification and Statistical Analysis

The raw data generated during the study are available as Supporting Information. Data were analyzed using R 4.4.0 [32]. Alpha was set to 0.05. We used a generalized linear mixed‐effect model (R package: lme4 [33]) with the dependent variable being binomial: 1 = congruent association (i.e., between higher pitch and white, and lower pitch and black, as reported in humans), 0 = incongruent association between pitch and luminance. We ran an Akaike information criterion (AIC)‐based model selection to determine the minimum adequate model, with predictors including the trial (from 1 to 16), the pitch (higher or lower), the position of the white area (left or right), and their interactions. We ran an analysis on the selected model using the R package emmeans [34]. The graph was generated using ggplot2 [35].

## 3 Results

**FIGURE 2:**
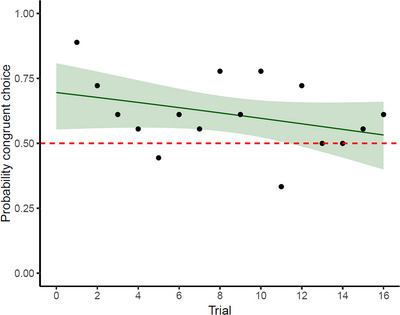
Probability of displaying the hypothesized crossmodal association across 16 testing trials, based on the door chosen by the tortoise. Each point represents group performance; the shaded area around the line (marked as green) represents the confidence interval; the dashed line (marked as red) indicates chance level (i.e., probability of 0.5).

Analysis of the minimum adequate model (based on the AIC) revealed that tortoises relied on a congruent association for which they chose more often the white door when hearing the higher‐pitch sound, and the black door when hearing the lower‐pitch one (prob(congruent association) = 0.612, SE = 0.03, *z* = 3.755, *p* < 0.001). This effect remained consistent across trials. Regarding a potential effect of experience with unrewarded trials during the test, and the issue of plasticity of crossmodal associations, we observed a trend for a decrease in pitch−luminance association during the test (Figure 2), although not statistically significant (effect of trial: β = −0.436; SE = 0.026; *z* = −1.647; *p* = 0.1).

## 4 Discussion

Our results show that Hermann's tortoises spontaneously associate a higher‐pitched sound with a light‐colored object (white panel) and a lower‐pitched sound with a dark‐colored object (a black panel), similar to what humans [4, 23] and chimpanzees [4] do. We interpret these findings within the framework of crossmodal correspondences, as the tortoises displayed a directional bias that mirrors the structured associations observed in adult humans [4, 23]. The presence of this pitch−luminance correspondence in a reptile suggests that the ability to link auditory and visual stimuli may have deep evolutionary roots, potentially serving adaptive functions across diverse ecological contexts or emerging from general advantages of sensory integration [36]. For instance, crossmodal associations may stem from shared processing of intensity features, with lightness and loudness potentially encoded by the same brain structures dedicated to magnitude [36, 37]. However, the very notion of amodal sensory dimensions remains debated, and such effects may also reflect parallel but distinct, modality‐specific mechanisms that are conceptually or functionally linked [38]. Together with mounting evidence of crossmodal associations in different taxa [4, 5, 6, 7, 8, 9], our results support the idea of the widespread presence of spontaneous crossmodal associations reflecting natural co‐occurrences in the environment and possibly present early in life [18, 39]. This challenges the idea that such associations are necessarily the product of language‐related or human‐specific ecological pressures. According to this view, the fact that the majority of human languages use terms like “high” and “low” to describe both pitch and luminance could reflect an inherent predisposition to link these modalities, rather than language itself being the origin of the association.

However, it remains unclear why other species, such as baboons and chickens, did not show similar crossmodal associations in comparable tests. Methodological factors and previous experience may explain these discrepancies. For example, chicks might have been overly exposed to high pitches and lightness during rearing, which could have influenced their responses. Additionally, the auditory stimuli used for testing chicks were the same as those for primates and may not have been ecologically relevant to birds, whose most sensitive auditory range is between 600 and 2500 Hz [24]. In the case of baboons, it has been suggested that attention issues or difficulty discriminating the auditory stimuli, coupled with their extensive experience with similar training protocols and visual tasks, could have affected performance [23]. This is consistent with the notion of spontaneous associations updating with experience [9, 24]. Previous studies in which animals were tested for other instances of crossmodal associations in extinction [8, 9] found that the effect diminished over repeated unrewarded trials, eventually reaching chance level. This shows how crossmodal associations tuned on specific pairings have a potential for flexibility and plasticity. Such flexibility is crucial for this mechanism to remain advantageous, enabling the subject to switch to a different strategy if their prior proves unhelpful [9]. It could also be based on frequency of exposure [24, 40]. It is also possible that pitch−luminance crossmodal associations genuinely do not exist in these species [23]. Directly retesting these species with paradigms adapted to their sensory and ecological needs will be crucial to disentangle the effect of experience and methodological influences from true interspecies variability [41]. Understanding the phylogenetic development of crossmodal correspondences is essential for advancing our knowledge of sensory processing, cognitive evolution, and the extent to which such perceptual associations are shared across different species. For instance, while crossmodal correspondences may contribute to multisensory integration, they can also occur independently of it [42], underscoring the importance of studying them in their own right.

Future studies could explore whether nonhuman species show similar crossmodal associations across other auditory and visual information. While our results show that tortoises spontaneously associate pitch with lightness in a manner comparable to humans, it remains to be tested whether they, like humans [43], also respond to other dimensions such as loudness or brightness. Additionally, although our study did not address whether crossmodal correspondences operate on a relative basis, this remains an important open question. Given that such correspondences are thought to reflect shared mechanisms across species, and that in humans they are consistently relative [44], it is reasonable to hypothesize that they function similarly in nonhuman animals. This provides a strong basis for future research. Supporting this view, there is already promising evidence that animals, including domestic chicks, apply relative processing rules in other domains—such as spatial–numerical associations [45]—suggesting that relative mappings may be a general principle of animal cognition, and likely extend to crossmodal correspondences as well.

## Author Contributions

Conceptualization, M.L. and E.V.; Methodology, M.L., B.M., G.S., and E.V.; Validation, M.L., B.M., and E.V.; Formal analysis, M.L. and E.V.; Investigation, M.L. and B.M.; Resources, M.L., G.S., and E.V.; Data curation, M.L. and B.M.; Writing – original draft, M.L.; Writing – review and editing, B.M., G.S., and E.V.; Visualization, M.L., B.M., and E.V.; Supervision, M.L., G.S., and E.V.; Project administration, M.L., G.S., and E.V.; Funding acquisition, M.L. and E.V.

## Conflicts of Interest

The authors declare no competing interests.

## Supporting information




**Supplementary Materials**: nyas70063‐sup‐0001‐SuppMatt.xlsx

## Data Availability

The data that support the findings of this study are available in the Supplementary Material of this article.
